# A clinical study on the application of three-dimensionally printed splints combined with finite element analysis in paediatric distal radius fractures

**DOI:** 10.3389/fped.2025.1559762

**Published:** 2025-04-03

**Authors:** Cheng Xu, Lefeng Wang, Meng Zhang, Xiao Li, Kuang Li

**Affiliations:** Limb Reconstruction and Pediatric Orthopedics Department, Shandong First Medical University, Tai'an, China

**Keywords:** forearm fractures, paediatric orthopaedics, biomechanics, 3D printing, finite element analysis, personalised medicine

## Abstract

**Purpose:**

This single-centre randomised clinical trial assessed the clinical efficacy and patient satisfaction of 3D-printed splints optimised via finite element analysis (FEA) for pediatric distal radius fractures.

**Methods:**

This retrospective study included 56 children diagnosed with forearm fractures at our hospital between August 2023 and August 2024. Those who underwent traditional U-shaped forearm plaster immobilisation were compared with those who received a customised 3D-printed splint. FEA was conducted based on the biomechanical characteristics of the forearm; the splint structure was optimised based on the analysis results and created via 3D printing. Outcomes were evaluated using the Patient Satisfaction Questionnaire and Wong-Baker Faces Pain Scale–Revised. Forearm function was evaluated using the Mayo Wrist Score and radiological outcomes. A power calculation was not performed due to the exploratory scope and resource limitations of this preliminary study.

**Results:**

The treatment costs significantly differed between the two groups (*p* = 0.001). In the Patient Satisfaction Questionnaire, the hot and sweaty item showed no significant difference (*p* *=* 0.089), whereas the last week's comfort (*p* *=* 0.001), first applied comfort (*p* *=* 0.004), weight (*p* *=* 0.001), itchiness (*p* *=* 0.015), smell (*p* *=* 0.003), and overall satisfaction items significantly differed between the two groups (*p* *=* 0.004). A comparison of the Mayo Wrist Score showed a statistically significant difference between the two groups after external fixation removal (*p* *=* 0.044). There were no significant differences between the two groups in terms of the palmar tilt angle (*p* = 0.196), ulnar deviation angle (*p* = 0.460), or height of the radial styloid (*p* = 0.111).

**Conclusion:**

Both 3D-printed splint and plaster cast fixation methods can effectively treat distal radial fractures in children, but the 3D-printed splint showed superior patient acceptance.

## Introduction

Forearm fractures are one of the most common fractures in children, comprising more than 40% of all paediatric fractures, with approximately three-quarters occurring at the distal radius (1, 2), and greatly affecting the intricate structures of the wrist and elbow joints (3). Currently, post-reduction fixation relies primarily on plaster casts, splints, and high-polymer materials (4). However, these materials often have poor fit, high complication risks, and heavy reliance on physician expertise. Additionally, patients' rehabilitation experiences are frequently affected by bulky, poorly ventilated splints, and less experienced physicians may inadvertently cause unnecessary discomfort or poor fixation. For stable pediatric distal radius fractures, consensus guidelines support immobilisation limited to the wrist for 3–4 weeks, as prolonged immobilisation may impair functional recovery (3, 5).

Owing to individual differences among children and their high activity levels, traditional bulky plaster casts or pre-fabricated polymer external fixation devices often fail to conform well to the child's forearm. This can lead to skin injury or inadequate immobilisation. Studies have indicated that anatomically shaped splints are effective in treating fractures, and custom three-dimensionally (3D) printed splints with ventilated structures improve comfort (6). For example, Hua et al. (7) used biomechanical analyses to compare the stress distribution of various splint types and concluded that anatomically shaped splints are the most effective for fracture treatment. Chen et al. (8) designed a 3D-printed forearm splint composed of two sections fastened with Velcro straps, which enable adjustment as the swelling subsides. Lazzeri et al. (9) designed orthoses using forearm skin data and 3D printing technology. Meanwhile, Sedigh et al. (10) designed a machine-learning model that enables the integration of artificial intelligence with 3D scanning to enhance the fit of pre-fabricated splints. However, most existing models of forearm splints based on 3D printing lack biomechanical analyses of stress and deformation or verification of their protective effects in clinical trials (8).

Finite element analysis (FEA) techniques can assist clinicians in better understanding the biomechanical characteristics of biological tissues and external fixation (11). In this study, we used a UScan scanner (UnionTech 3D Co., Ltd., Shanghai, China) to capture forearm model data, which were imported into Meshmixer (Autodesk, Inc., Ltd., San Rafael, CA, USA) and Magics software (Materialize NV., Ltd., Leuven, Belgium) to design a forearm fracture splint. FEA was subsequently applied to evaluate the splint's protective effect on the fracture site under external forces, providing guidance on ventilation, weight, and volume optimisation of fracture splints. Clinical trials confirmed the effectiveness and reliability of this approach on splint design, potentially improving fracture recovery with the assistance of a personalised, stable, and effective 3D-printed splint. In this study, we aimed to explore the feasibility of combining 3D printing technology and FEA to design and create forearm splints as well as to demonstrate their reliability through clinical trials.

## Materials and methods

### Patients

Between August 2023 and August 2024, 56 patients with distal radial fractures were treated at our hospital. The inclusion criteria were age of 5–13 years and having isolated radius fractures, all patients had closed, non-displaced, or minimally displaced distal radius fractures (AO/OTA classification 2R3-M/2R3-U1) confirmed via radiography. Patients with associated ulnar fractures, other fractures or trauma, isolated radial fractures with associated nerve injury, a history of acute or chronic diseases that may affect bone development, and a history of reactions or hypersensitivity related to materials used for splint production were excluded. This study strictly adhered to the inclusion and exclusion criteria. Eligible patients were assigned unique numbers and randomly grouped using a computer-generated sequence in an Excel spreadsheet. The patients were grouped according to the fixation used: plaster cast group (*n* = 28), those who used traditional U-shaped plaster cast fixation; 3D-printed splint group (*n* = 28). The general characteristics of the two groups did not statistically differ (Table 1).

**Table 1:** Comparison of general information between two groups of patients.

Group	Age (years)	Gender	
		Male	Female
3D printed splint group (*n* = 28)	10.67 ± 2.60	22	6
Plaster cast group (*n* = 28)	8.86 ± 2.77	22	6
t-value	1.692	0.000	0.000
*P*-value	0.096	1.000	1.000

### Treatment procedure

All procedures were performed at a single clinical centre by the same surgical team. Patients were positioned upright, with their forearms maintained in the functional position. Both patient groups underwent manual reduction. The key difference was that patients in the plaster cast group received manual reduction immediately following their visit, whereas those in the 3D-printed splint group underwent manual reduction and had the orthosis applied after the printing process was completed.

For the traditional U-shaped plaster cast fixation, manual reduction was performed first, followed by the reduction of the fracture ends. Orthopaedic synthetic bandages were moistened with water, and cotton padding was applied on the outside. The plaster bandages were then wrapped in a U-shape around the forearm, securing the fracture ends with the bandages. After the bandages hardened, the cast was complete.

**Figure 1:**
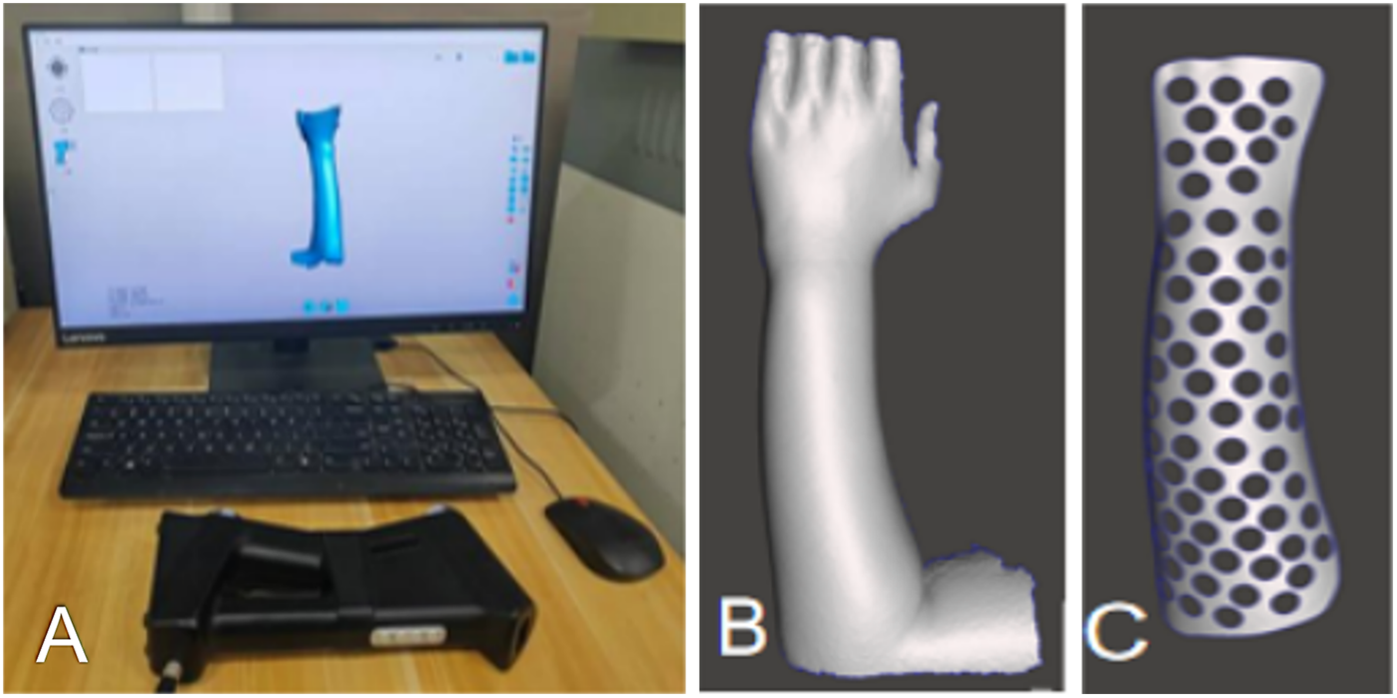
**(A)** Skin surface data collection using unionTech 3D white-light dual-vision handheld scanner. **(B,C)** Model construction using Meshmixer.

**Figure 2:**
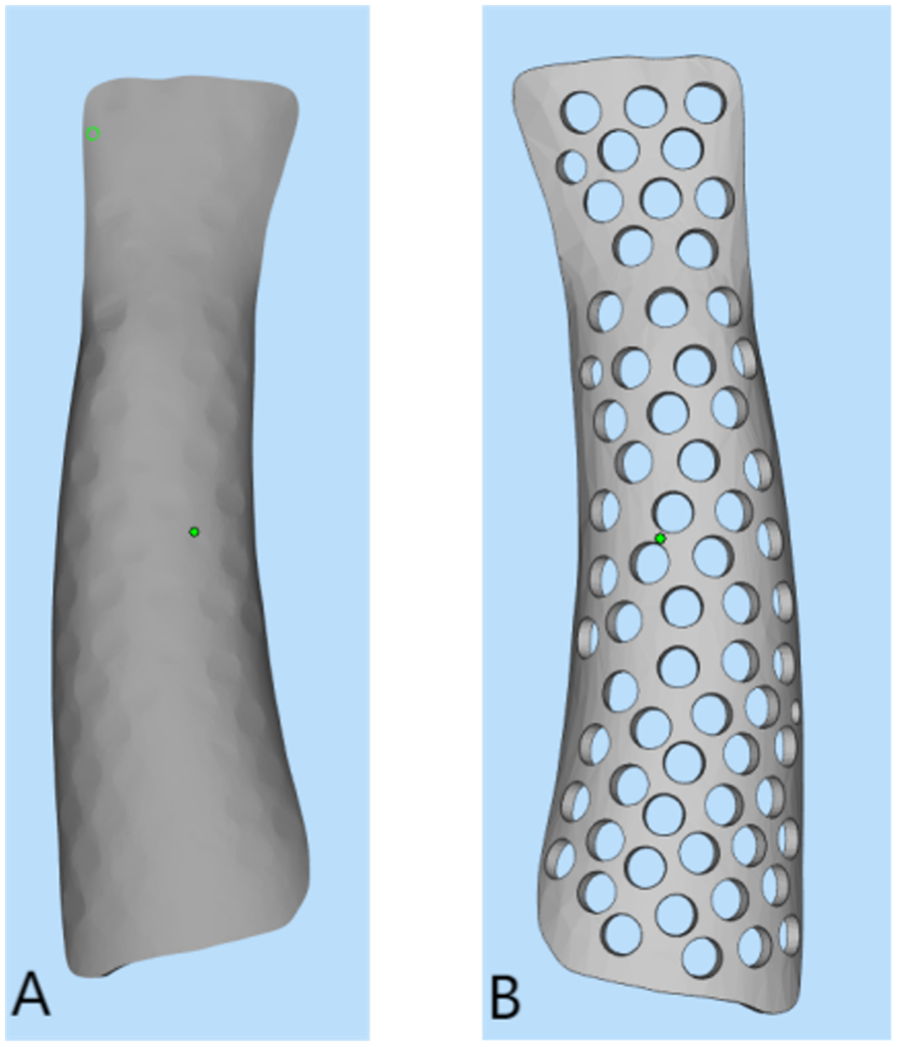
**(A)** Forearm splint model built by meshmixer. **(B)** The model optimised by Magics.

**Figure 3:**
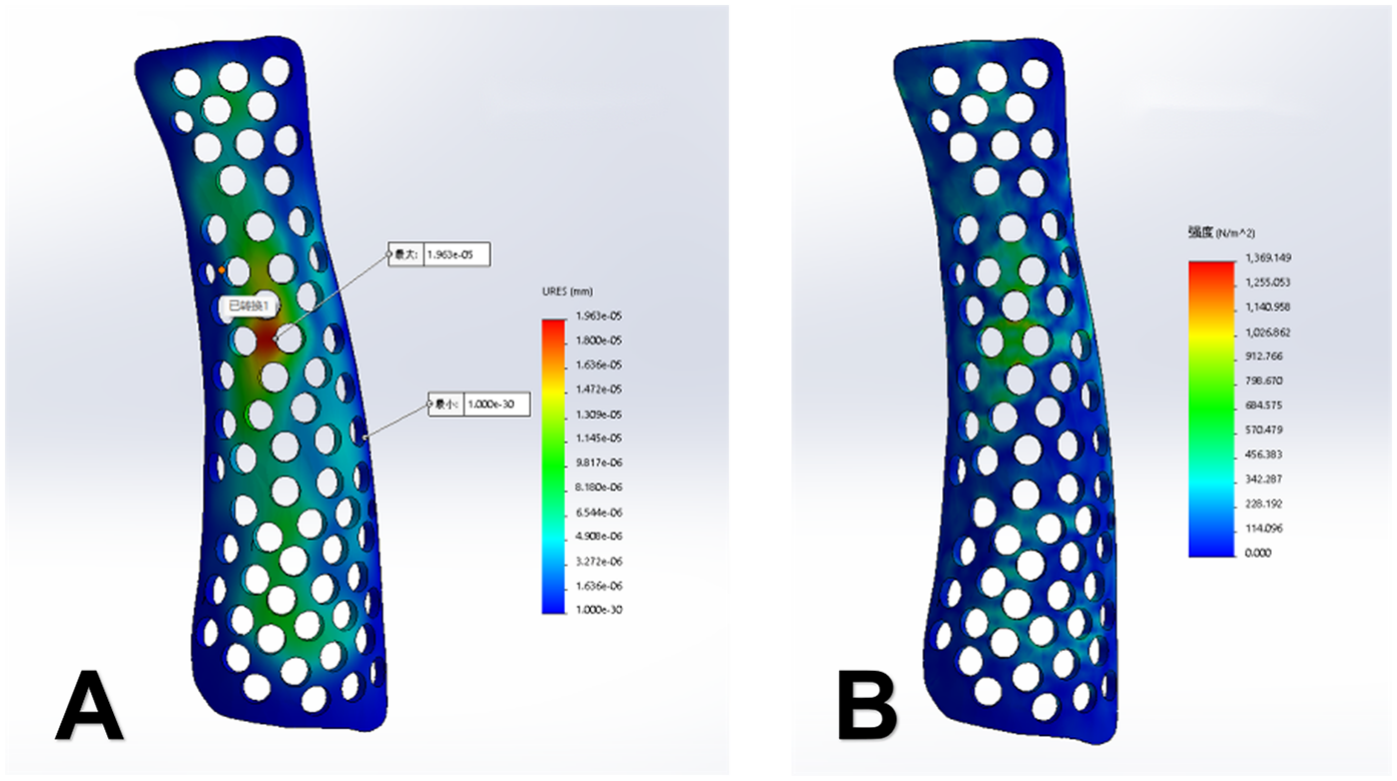
**(A)** Displacement contour map of the splint (displacement, 0.002 mm). **(B)** Stress distribution contour map (maximum stress, 1.36 kPa).

**Figure 4:**
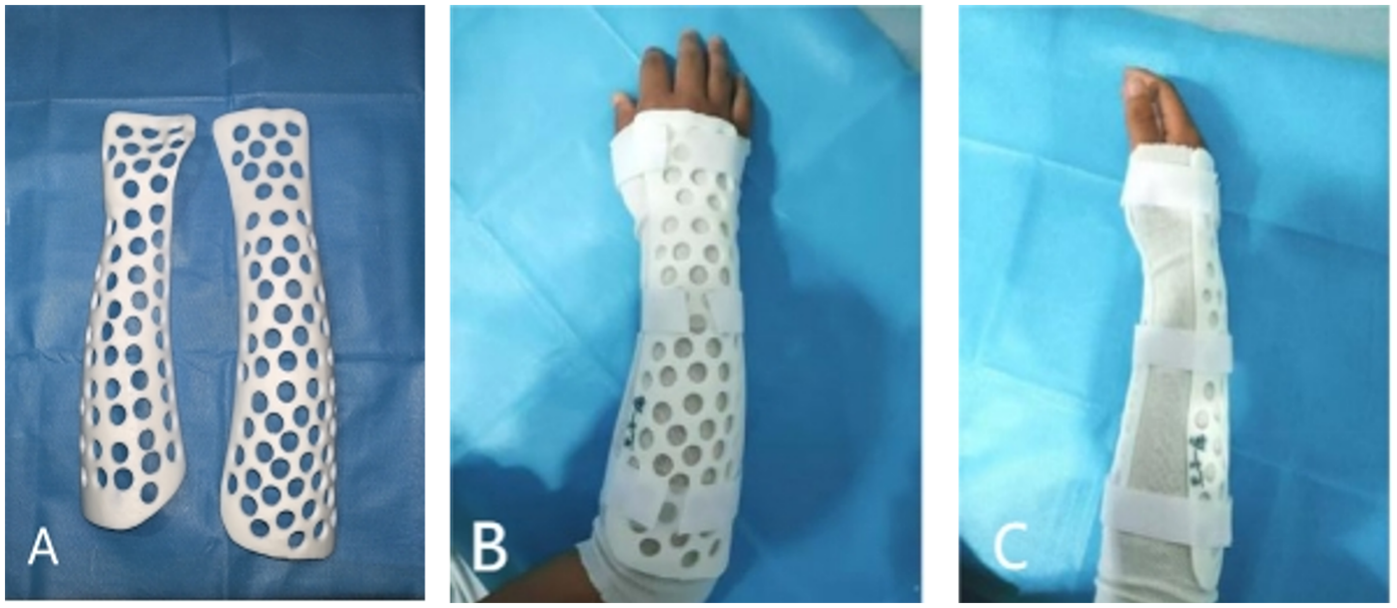
**(A)** Three-dimensionally printed splint made of acrylonitrile butadiene styrene. **(B,C)** Photographs of the arm of the patient wearing the splint.

For the 3D-printed splint, a UnionTech 3D white-light dual-vision handheld scanner (UnionTech 3D Co., Ltd.) was used to scan both forearms of the patients, capturing 3D models of the functional position on both the palmar and dorsal sides. UScan software (UnionTech 3D Co., Ltd.) was used to generate a 3D model of the skin surface, which was then exported in STL format. The exported data were subsequently imported into Meshmixer software (Autodesk, Inc., Ltd.), where the surface treatment function was used to obtain the surface of the splint (Figure 1). The skin surface data from the patient's forearm were imported into Magics software version 22.0 (Materialize NV, Ltd.), where processes of thickening and smoothing were applied to construct the forearm splint model. Defects were repaired to ensure model completeness. Subsequently, the data was imported into SolidWorks 2023 (Dassault Systèmes, Waltham, Massachusetts, USA). Here, Non-Uniform Rational B-Spline (NURBS) surface models were created using polygon modelling and precise surface fitting. The preparation time for this model depends on the equipment's performance and scanning accuracy, typically ranging from 1 to 2 h. A clinician trained in finite element analysis (FEA) performed this procedure. Once the NURBS surface model was completed, optimisation was performed to improve the model fit. The optimised digital model file was imported into Magics software for slicing, and the splint was printed. All testing methods followed the ASTM standards, as detailed in Table 2. The collected data were imported into Meshmixer software, where the surface data of the forearm splint were cut out and a 3D model was generated (Figure 2). The design of the 3D-printed splint should clearly define the front, rear, and contour boundaries and determine the regions for optimisation and non-optimisation. Uniform holes were created within a 5-mm boundary. The splint was processed using the Meshmixer plugin for material reduction, and the optimised model is presented in Figure 2. FEA was performed to evaluate the stability and protective effect of the 3D-printed splint on the fracture site. Boundary constraints were applied to the splint, and a uniform downward force of 100 N was applied to its surface. The stress distribution and displacement of the splint are presented in Figure 3. In this study, a photopolymerisation moulding technique was used. Depending on the model size, the printing process took two to three hours. After printing, the splints were cleaned with alcohol, cured under UV light, and polished to enhance comfort. Finally, the outer layer of the splint was wrapped with a tubular polymer elastic bandage. The 3D-printed splint material used a UV-cured resin similar to acrylonitrile butadiene styrene, with its post-cured mechanical properties assigned based on relevant literature. The splint was dismantled, and the edges were polished to prevent skin damage. A Velcro strap was added to the 3D-printed splint to improve patient comfort and adjustability. In this study, the outer layer of the splint was wrapped with a tubular polymer elastic bandage to improve the fit and enhance patient comfort and satisfaction. The processed splint and photographs of the arm of the patient wearing it are presented in Figure 4.

**Table 2:** Mechanical properties of the resin after 90-minute UV curing tested according to ASTM standards.

Measurement	Test method	Value (90-minute UV curing)
Hardness	ASTM D 2240	83
Flexural modulus	ASTM D 790	2,692–2,775
Flexural strength	ASTM D 790	69–74
Tensile modulus	ASTM D 638	2,189–2,395
Tensile strength	ASTM D 638	27–31
Elongation at break	ASTM D 638	12–20%
Impact strength J/m	ASTM D 256	58–70
Heat deflection	ASTM D 648 @66PSI	52
Density		1.16

### Outcome assessment and statistical analyses

The treatment duration was four weeks, after which the plaster and splints were removed. The Wong-Baker Faces Pain Scale–Revised (FPS-R) was used, as it is suitable for children, the elderly, and those with lower levels of education, and it can even be used for patients who have difficulty expressing themselves (12). The FPS-R scores after immobilisation and two weeks after the fracture were used to evaluate wrist pain caused by distal radius fractures. The Patient Satisfaction Questionnaire (Table 3) was constructed using terminologies that can be easily understood by the paediatric population, was not age-dependent, and was generally completed by both patients and parents (5). Radiographic healing assessments were performed according to the anatomical standards of the wrist joint, including palmar tilt angle, ulnar variance, and radial styloid height. Functional recovery was evaluated using the Mayo Wrist Score (13). Data from both groups were compared using independent sample *t*-tests, and *p* < 0.05 was considered statistically significant. The collected clinical data were processed and analysed using SPSS version 21.0 (IBM Corp., Armonk, NY, USA) (14). Prism 10 (GraphPad, La Jolla, San Diego, USA) was used to draw shapes.

**Table 3:** Patient satisfaction questionnaire.

Question	Response				
	1	2	3	4	5
In the last week has your cast been comfortable?	Want it removed	Irritating	Fairly comfortable, occasional irritation	Comfortable	Very comfortable
When your cast was first applied.	Uncomfortable	Took up to a week to become comfortable Moderately heavy.	Fairly comfortable after a few days	Took 1–2 days to become comfortable	Very easy to get used to
Weight of cast	Heavy cast. Difficult to use arm	Limited multiple activities.	Mildly heave limited, several activities	Fairly light	Light cast. Didn't interfere with activities
Itchiness	Very itchy	Frequent itch but tolerable	Sometimes itchy	Rarely itchy	No itch
Hot and sweaty	Very hot. Wanted cast removed	Hot feeling worrying Complained a lot.	Often hot Mild distress	Hot at times but tolerable	Well tolerated
Smell	Distressing smell	Continual mild odour	Smell after hotday	Occasional smell	None
Overall satisfaction	Awful, intolerable	OK, not as easy as imagined	Good overall comfort	Very comfortable	Excellent recommend to friends

## Results

### I. Study population and completion status

This study included a total of 56 patients, with 28 in the plaster cast group and 28 in the 3D splint group. All participants successfully completed the designated treatment plan and followed up for four weeks (4). The two groups were balanced in baseline characteristics such as age, gender, and injury type, meeting the design requirements of a randomised controlled trial (Table 1, *p* > 0.05).

### II. Patient satisfaction evaluation

The standardised Patient Satisfaction Questionnaire revealed significant differences (*p* < 0.05) in several areas: comfort during the last week (*p* = 0.001), comfort when first applied (*p* = 0.004), weight (*p* = 0.001), itchiness (*p* = 0.015), smell (*p* = 0.003), and overall satisfaction (*p* = 0.004). No significant difference was found in heat and sweating sensation (*p* = 0.089).

### III. Treatment costs and operational efficiency

There was a significant difference in treatment costs between the two groups (*p* = 0.001), with the 3D splint group incurring approximately 48% higher costs per case (Table 4). Additionally, there was a significant difference in operation time (*p* = 0.001), with the plaster cast averaging 0.72 h and the 3D splint averaging 2.85 min, limited by 3D printing speed and model optimisation time (Table 5).

**Table 4:** Plaster cast group and 3D-printed brace group cost comparison.

Cost components	3D splint group	Plaster cast group
Treatment costs (CNY)	362.21 ± 13.81	248 ± 0.00
Material costs	93.18 ± 10.31	56 ± 0.00
Labor cost	192 ± 0.00	192 ± 0.00
Equipment and maintenance costs	79.03 ± 3.50	0.00

**Table 5:** Comparison of clinical data between the plaster cast group and the 3D printed splint group.

Clinical assessment parameters and outcomes	3D splint group	Plaster cast group	*p*-value
Patient satisfaction questionnaire			
Last week comfort	3.32 ± 1.25	1.96 ± 1.00	0.001
First applied comfort	2.39 ± 1.13	1.61 ± 0.74	0.004
Weight	3.11 ± 1.40	4.64 ± 1.97	0.001
Itchiness	3.57 ± 0.88	3.00 ± 0.81	0.015
Hot and sweaty	2.46 ± 1.374	3.07 ± 1.25	0.089
Smell	2.57 ± 1.32	3.64 ± 1.25	0.003
Overall satisfaction	2.71 ± 1.27	1.57 ± 0.84	0.004
Wrist function (MMWS)			
Mayo wrist score	89.64 ± 8.71	88.57 ± 6.22	0.044
Excellent	14 (50.0%)	13 (46.4%)	
Good	11 (39.2%)	14 (50.0%)	
Fair	3 (10.7%)	1 (3.6%)	
Poor	0 (0.00%)	0 (0.00%)	
Pain (FPS-R)			
Score	3.18 ± 1.79	4.00 ± 1.30	0.049
Treatment costs(CNY)	197.21 ± 13.81	248 ± 0.00	0.001
Treatment time(hours)	2.85 ± 0.93	0.72 ± 0.34	0.001
Radiological outcomes			
Palmar tilt angle	27.41 ± 1.80	26.80 ± 1.74	0.196
Ulnar variance	35.07 ± 2.95	34.48 ± 2.97	0.460
Radial styloid height	11.83 ± 0.46	12.01 ± 0.38	0.111

### IV. Functional recovery and complications

No significant difference was observed in excellent/good wrist function rates between the groups (*p* = 0.621), with 82.1% in the plaster cast group and 85.7% in the 3D splint group. Complications included two cases (7.1%) of joint dysfunction in the 3D splint group, which manifested as limited flexion and resolved after 4 weeks of rehabilitation training, and three cases (10.7%) of skin irritation at the ulnar styloid process in the plaster cast group, which resolved within 2 weeks after removal. A significant difference was noted in pain scores (*p* = 0.049).

### V. Comprehensive evaluation results

#### Subjective patient evaluations

The 3D splint group demonstrated significant improvements in patient satisfaction (*p* < 0.01) and Mayo Wrist Score (*p* = 0.007), particularly in terms of convenience for daily activities and ease of hygiene maintenance.

#### Objective evaluation metrics

**Figure 5:**
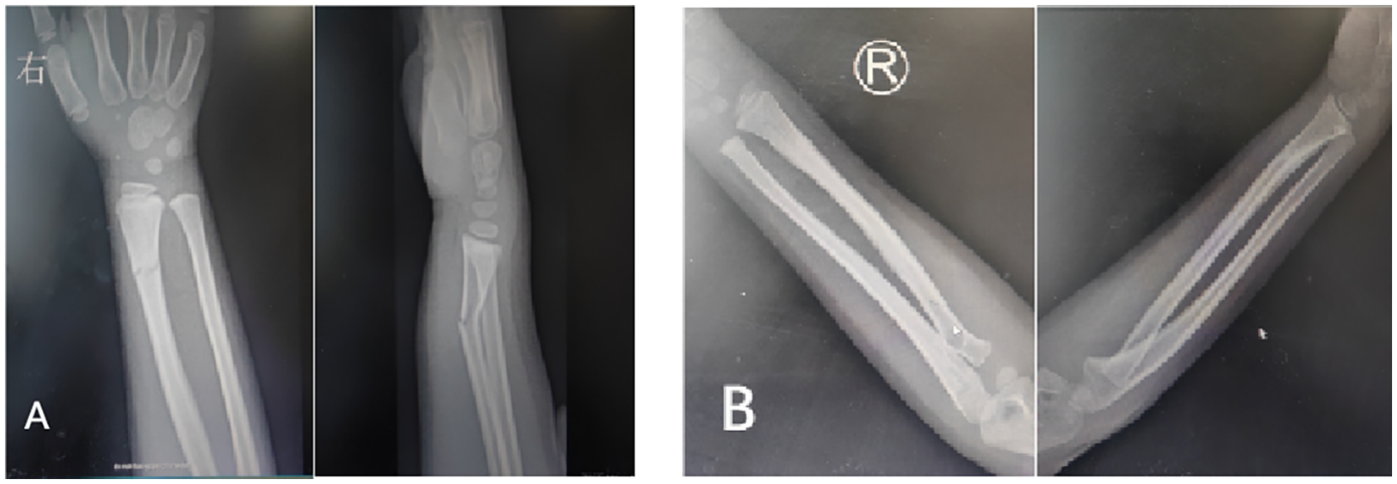
**(A)** X-ray after patient injury. **(B)** X-ray when patient uses 3D-printed splint for 4 weeks.

Radiological outcomes (Palmar Tilt Angle, Ulnar Variance, Radial Styloid Height) were comparable between the two groups (*p* > 0.05), with no significant differences observed in fracture healing time or alignment accuracy. All fractures achieved radiographic union by 4 weeks, defined as bridging trabeculae across the fracture site on anteroposterior and lateral views (Figure 5).

## Discussion

This study showed that 3D-printed splints were more effective than the traditional method (plaster fixation) for paediatric distal radius fractures. Plaster and splint external fixation techniques have been widely used in the non-surgical treatment of paediatric forearm fractures and can successfully achieve healing at the fracture site (4). However, owing to their lack of breathability, pressure imbalances, and other drawbacks, complications such as skin diseases and compartment syndrome may arise, negatively affecting the patient's quality of life and reducing patient compliance. 3D-printed splints offer substantial advantages such as comfort, lightweight design, and ease of use. Furthermore, the 3D-printed splints used in this study are removable, which can improve skin hygiene and reduce the risk of skin pressure sores and ulcers. This significantly affects patient experience, especially in children and elderly individuals who require regular skin monitoring (15, 16). Since this experiment involves multiple steps, the total time required is primarily determined by the size of the orthosis. Based on our experience, it typically takes approximately 4–5 h. In clinical practice, some parents, eager to alleviate their child's pain quickly, may opt for a fast procedure such as plaster cast fixation. The parents' main concerns include reduced limb movement due to fears of sweating or getting wet, limited rehabilitation activities, and the inability to remove or inspect the skin beneath the splint for ulcers or pressure sores (17). Janzing et al. (18) suggested that not considering early inflammation and swelling when designing a splint could result in loss of fracture alignment. To solve this problem, the splint was adjusted to fit the surface of the healthy contralateral limb, and the splint shape was fine-tuned to ensure a gap between the splint and the skin surface. Velcro straps were added to both sides of the splint to allow adjustment for swelling during the inflammation phase.

With the help of Magics, we pre-designed the 3D-printed splint to conform to the normal shape after manual reduction and maintain the reduction of the fracture ends by tightening the Velcro straps, and these characteristics demonstrate the superiority of the 3D-printed orthoses. The 3D-printed splint achieved comparable radiological outcomes (e.g., palmar tilt angle) to traditional casts due to its personalised design, which conforms to the reduced fracture anatomy. The adjustable Velcro straps allowed dynamic immobilisation, maintaining alignment while accommodating swelling. Lightweight materials and ventilated structures likely contributed to reduced discomfort without compromising stability. In addition, the design data of 3D-printed splints can be stored, making it easy and convenient to reprint the splint. Research on the use of wooden plastic splints as an alternative to plaster splints exists, which has advantages in terms of weight and comfort. While wooden splints have environmental advantages, 3D-printed splints offer several unique benefits: They provide a precise anatomical fit, enhancing both immobilisation and patient comfort. Their lightweight, breathable, and adjustable designs improve patient compliance and overall experience. Through optimised topology, such as the use of uniform holes, material waste is significantly reduced. Future research should focus on comparing the environmental impact of 3D printing resins with traditional materials, while developing the reliability of biodegradable resins in clinical applications, to further evaluate their sustainability.

Along with ensuring clinical treatment efficacy and safety, cost and printing time are two important factors in the clinical application of 3D printing solutions. According to existing research, the advantages of 3D-printed splints outweigh those of plaster casts and low-temperature thermoplastic techniques. These advantages include better fit, aesthetics, lightweight design, and improved medical rehabilitation and skin care. In the present study, the cost of 3D printing was higher than that of plaster fixation. As indicated in Table 4, the material cost for the 3D-printed splint group primarily involves the cost of resin, which varies for each patient based on the size of the orthosis. For the plaster cast group, the cost is fixed at 50 RMB for orthopaedic synthetic plaster. Labour costs are identical for both groups, as there are no outsourced personnel in this study, and they are based on the hospital's stipulated fee for plaster cast fixation. Equipment costs differ significantly: the plaster cast group incurs no equipment maintenance fees, while the 3D printing group bears costs for equipment depreciation and maintenance. These factors contribute to a notable cost difference between the two groups. However, our hospital's early adoption of 3D printing has allowed for the amortisation of these costs. Looking ahead, as resin prices decline, equipment is upgraded, and 3D printing technology advances, we anticipate further reductions in expenses. Additionally, the lower incidence of complications in the 3D printing group potentially reduces overall treatment costs. The primary factor affecting treatment operation time is the 3D printing duration, dependent on the printer's speed. In clinical settings, the main costs associated with 3D printing stem from the necessary equipment, such as scanners and 3D printers. With the increasing adoption of 3D printing, its costs are expected to decrease considerably.

Additionally, the learning curve for 3D-printed splints is long (approximately 20 days), requiring five hours of training daily, including 2.5 h of theoretical class and 2.5 h of practical class, which makes it difficult to devote considerable time to learning such technologies in clinical practice (19). This also applies to FEA, which requires time investment. Consequently, many hospitals have hired specialised personnel when introducing 3D printing technology, further increasing costs and creating challenges for the clinical application of 3D printing.

Many methods are used for treating forearm fractures clinically; however, biomechanical research is the foundation for determining treatment plans. Biomechanics is a discipline that involves quantitative studies of mechanical problems in organisms based on human anatomy, physiology, and mechanical theories and methods. This study indicates that FEA plays a crucial role in biomechanical research tasks and has been successfully applied for many years in assessing the effects of external loads on biological tissues. Urendes et al. (20) designed a passive upper-limb exoskeleton based on biomechanical characteristics for rehabilitation after upper-limb nerve and muscle injuries. They primarily analysed the impact of external loads on the entire splint; therefore, these simplifications do not reduce the validity of the results. With the integration of computer-aided technology, 3D printing, and 3D scanning with the medical field, 3D-printed external fixation splints represent a trend in the future treatment of bone fractures (21). With this development, 3D printing has become more common in the treatment of upper-extremity fractures. A 3D-printed cast offers excellent features that improve patient care and satisfaction (22). Currently, only a few 3D-printed products are available in the market, partly because of the relatively high production cost and the lack of large-scale clinical trials verifying the protective effect and overall biomechanical performance of 3D-printed splints. Research has shown that the main reason for fracture re-displacement is the shear force caused by muscle traction and external loading.

Topology optimisation is a method that optimises the material distribution by seeking the best arrangement under given load and boundary conditions while meeting specific performance requirements. The goal of this study was to minimise the volume of the splint while ensuring optimal stiffness. The final hollow design was selected by comparing the strength and clinical convenience of splints with different patterns and thicknesses. In the model design, circular holes with a thickness of 4 mm were created uniformly within a 5-mm boundary region. In this study, FEA was used to apply a 100-N uniform external force to the splint, and its maximum displacement and stress distribution were calculated. The results showed that splint displacement of 0.002 mm and a maximum stress concentration of 1.36 kPa, and there was no stress concentration at the fracture site. This suggests that the new 3D-printed splint can effectively protect the fracture site during accidental impact. The 3D-printed external fixation splint designed in this study is lightweight, easy to shape, and comfortable to wear, aiding in post-fracture rehabilitation and healing.

The clinical data showed no significant difference in the prognoses of the 3D-printed splint and plaster cast groups during the rehabilitation process, which is consistent with the findings of other studies. A 3D-printed cast was functionally non-inferior to the traditional splint while providing a water-resistant, lightweight, and breathable alternative (15). Graham et al. (15) designed an instant 3D-printed cast, and it was better than polymer orthosis, which is probably due to the more streamlined design and lighter structure of the instant 3D-printed cast than those of the polymer orthosis. With FEA support, this study achieved almost the same mechanical properties while remaining as lightweight as possible. However, a significant difference was observed in the Patient Satisfaction Questionnaire, which further supports the superiority of anatomically shaped splints. Furthermore, no skin damage higher than stage 1 according to the National Pressure Ulcer Advisory Panel/European Pressure Ulcer Advisory Panel classification was observed in either group, and no negative data were recorded. While the Mayo Wrist Score improvement was statistically significant, its clinical relevance requires further validation with larger cohorts. The modest effect size may reflect the short follow-up period or inherent limitations of functional scoring systems in capturing patient-centered outcomes like comfort.

This study had some limitations. This study focused on short-term outcomes (4 weeks post-treatment) to evaluate immediate efficacy and acceptance. Long-term follow-up (e.g., 6–12 months) will be incorporated in future work to address these aspects comprehensively. As 3D printing is a relatively new technology, it may possess a “cool factor” that could lead patients to subjectively favour the 3D-printed splint, potentially introducing both data and subjective bias during data collection. To mitigate these biases, future studies should consider volunteer blinding to obscure the type of splint received (3D-printed vs. traditional). This could help control for the novelty effect. Additionally, increasing the sample size and conducting multi-centre studies could further reduce data bias. Subjective biases are known to impact patient satisfaction scores. Future research should explore the feasibility and reliability of incorporating third-party evaluators. Moreover, incorporating wearable sensors to gather objective activity data and implementing double-blind evaluation mechanisms would enhance the credibility of the results. Further research is also needed to evaluate the safety and benefits of 3D-printed splints in orthopaedic patients comprehensively. Given that this was a single-centre study with a small sample size, well-designed randomised controlled multi-centre trials are essential to elucidate the clinical applications of this new technology.

## Conclusion

While both methods achieved equivalent fracture healing, 3D-printed splints demonstrated superior patient comfort and satisfaction. These advantages, combined with customisable design, position 3D-printed splints as a viable option for stable pediatric distal radius fractures where patient compliance and comfort are priorities. The 3D-printed splints providing a new method for the advancement of personalised medicine.

## Data Availability

The raw data supporting the conclusions of this article will be made available by the authors, without undue reservation.
